# *N*-Benzoyl dithieno[3,2-b:2′,3′-d]pyrrole-based hyperbranched polymers by direct arylation polymerization

**DOI:** 10.1186/s13065-017-0367-0

**Published:** 2017-12-21

**Authors:** Tam Huu Nguyen, Thu Anh Nguyen, Hoan Minh Tran, Le-Thu T. Nguyen, Anh Tuan Luu, Jun Young Lee, Ha Tran Nguyen

**Affiliations:** 1 Faculty of Materials Technology, Ho Chi Minh City University of Technology (HCMUT), Vietnam National University, 268 Ly Thuong Kiet, District 10, Ho Chi Minh City, Vietnam; 2 Materials Technology Key Laboratory (Mtlab), Vietnam National University-Ho Chi Minh City, 268 Ly Thuong Kiet, District 10, Ho Chi Minh City, 70000 Vietnam; 3 Department of Chemical Engineering, Sungkyunkwan University, Suwon, 16419 Republic of Korea

**Keywords:** *N*-benzoyl dithieno[3,2-b:2′,3′-d]pyrrole, 3-Hexylthiophene, Hyperbranched polymers, Direct arylation polymerization

## Abstract

**Background:**

Although poly(*N*-acyl dithieno[3,2-b:2′,3′-d]pyrrole)s have attracted great attention as a new class of conducting polymers with highly stabilized energy levels, hyperbranched polymers based on this monomer type have not yet been studied. Thus, this work aims at the synthesis of novel hyperbranched polymers containing *N*-benzoyl dithieno[3,23,2-b:2′,3′-d]pyrrole acceptor unit and 3-hexylthiophene donor moiety via the direct arylation polymerization method. Their structures, molecular weights and thermal properties were characterized via ^1^H NMR and FTIR spectroscopies, GPC, TGA, DSC and XRD measurements, and the optical properties were investigated by UV–vis and fluorescence spectroscopies.

**Results:**

Hyperbranched conjugated polymers containing *N*-benzoyl dithieno[3,23,2-b:2′,3′-d]pyrrole acceptor unit and 3-hexylthiophene donor moiety, linked with either triphenylamine or triphenylbenzene as branching unit, were obtained via direct arylation polymerization of the *N*-benzoyl dithieno[3,23,2-b:2′,3′-d]pyrrole, 2,5-dibromo 3-hexylthiophene and tris(4-bromophenyl)amine (or 1,3,5-tris(4-bromophenyl)benzene) monomers. Organic solvent-soluble polymers with number-average molecular weights of around 18,000 g mol^−1^ were obtained in 80–92% yields. The DSC and XRD results suggested that the branching structure hindered the stacking of polymer chains, leading to crystalline domains with less ordered packing in comparison with the linear analogous polymers. The results revealed that the hyperbranched polymer with triphenylbenzene as the branching unit exhibited a strong red-shift of the maximum absorption wavelength, attributed to a higher polymer stacking order as a result of the planar structure of triphenylbenzene.

**Conclusion:**

Both hyperbranched polymers with triphenylamine/triphenylbenzene as branching moieties exhibited high structural order in thin films, which can be promising for organic solar cell applications. The UV–vis absorption of the hyperbranched polymer containing triphenylbenzene as branching unit was red-shifted as compared with the triphenylamine-containing polymer, as a result of a higher chain packing degree. 
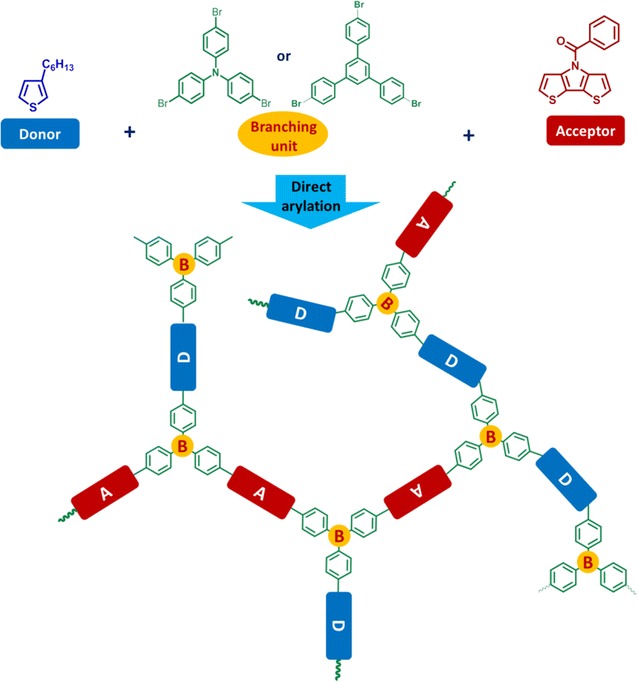

## Background

Conjugated polymers have received significant attention in fundamental and applied research owing to their interesting optical and optoelectronic properties. Thus, they have been used in many electronic applications such as light emitting diode (OLED), polymeric solar cells (PSCs), electrochromic devices, organic field-effect transistors (OFETs), chemo-and biosensors [1–4]. In these extensive applications, the donor–acceptor (D–A) type of conjugated polymers, consisting of both electron donor and electron acceptor substituents along the conjugated backbone with excellent electron mobility, broad absorption spectrum and properly matched energy levels, has generated significant interest in the field of PSCs [5–10]. Especially, conjugated polymers composed of various thiophene-based electron donating units have shown promising properties to be suitable as hole-transporting materials in electro-optical devices [11–13].

On the other hand, *N*-benzoyl dithieno[3,2-b:2′,3′-d]pyrrole belongs to a new class of dithieno[3,2-b:2′,3′-d]pyrroles incorporating *N*-acyl groups with highly stabilized energy levels, which have been studied for some years [14]. Evenson and Rasmussen [15] have reported for the first time the synthesis of the *N*-benzoyl dithieno[3,2-b:2′,3′-d]pyrrole and analogous monomers via copper-catalyzed amidation. *N*-octanoyl dithieno[3,2-b:2′,3′-d]pyrrole was further electropolymerized, resulting in poly(*N*-octanoyl dithieno[3,2-b:2′,3′-d]pyrrole) with a polymeric bandgap of 1.60 eV [15]. An *N*-substituted benzoyl dithieno[3,2-b:2′,3′-d]pyrrole was copolymerized with 4,7-dithieno-2,1,3-benzothiadiazole to give a polymer with a low band gap of 1.44 eV, the PSC of which had a power conversion efficiency (PCE) of 3.95% [16]. Poly(*N*-alkanoyl dithieno[3,2-b:2′,3′-d]pyrrole-*alt*-quinoxaline)s have been shown to afford PSCs with high open-circuit voltages and PCEs up to 4.81% [17]. More recently, Busireddy et al. [18] have reported the synthesis of a small molecule containing dithieno[3,2-b:2′,3′-d]pyrrole (DTP) and butylrhodanine as donor and acceptor moieties. PSCs fabricated from this donor material and [6]-phenyl-C71-butyric acid methyl ester as acceptor reached a PCE of 6.54% [18].

Hyperbranched conjugated polymers with highly branched molecular structure can effectively suppress aggregation and therefore are attractive due to good solubility and processability, low viscosity as well as facile one-pot synthesis and tunable electrical properties. Despite extensive research on the synthesis of hyperbranched conducting polymers in the past [19–21], in the last couple of years considerable effort has been put into the development of hyperbranched conjugated structures based on new compositional units. The Cu(I)-catalyzed azide–alkyne click reaction was used to synthesize an ethynyl-capped hyperbranched conjugated polytriazole [22]. Zhou et al. [23] employed Suzuki coupling polymerization to obtain hyperbranched polymers based on alkyl-modified 2,4,6-tris(thiophen-2-yl)-1,3,5-triazine and fluorene units with high molecular weights and enhanced two-photon absorption as compared with their unsubstituted analogues. The Suzuki polymerization was also used to one-pot synthesize a hyperbranched conjugated polymer bearing dimethylamino groups to be used as a PSC cathode interlayer [24]. Sen et al. [25] synthesized hyperbranched conjugated polymers based on 4,4′‐difluoro‐4‐bora‐3a,4a‐diaza‐*s*‐indacene (BODIPY) via Sonogashira cross coupling polymerization reactions. The polymers showed red shifts in absorption and emission maxima upon contact with toluene and benzene vapors. Very recently, hyperbranched thiophene-flanked diketopyrrolopyrrole (TDPP)-based polymers with narrow bandgaps were prepared by direct arylation polymerization method [26]. Knoevenagel condensation and Sonogashira coupling methods were used to synthesize different hyperbranched conjugated polymers, which were tested as chemosensors for detecting nitroaromatic compounds [27–29]. The base-catalyzed reactions between α,β-unsaturated ester and aldehyde was employed to synthesize hyperbranched conjugated polymers containing 1,3-butadiene repeating units and carboxylic ester side groups for sensing metal ion Fe^3+^ [30].

To the best of our knowledge, *N*-acyl dithieno[3,2-b:2′,3′-d]pyrrole-based hyperbranched conjugated polymers have not yet been studied. In this research, we present the synthesis of hyperbranched polymers having *N*-benzoyl dithieno[3,2-b:2′,3′-d]pyrrole and 3-hexylthiophene monomer units, linked with triphenylamine or triphenylbenzene as chain extender, via the direct arylation polycondensation [31]. Besides the role of branch-forming units, triphenylamine and triphenylbenzene are also typical donor moieties in conjugated polymeric materials for optoelectronic devices [32–37]. The optical and thermal properties and the nanostructures of the obtained hyperbranched polymers were characterized, and the effect of polymer aggregation on optical properties was investigated.

## Results and discussion

**Scheme 1:**
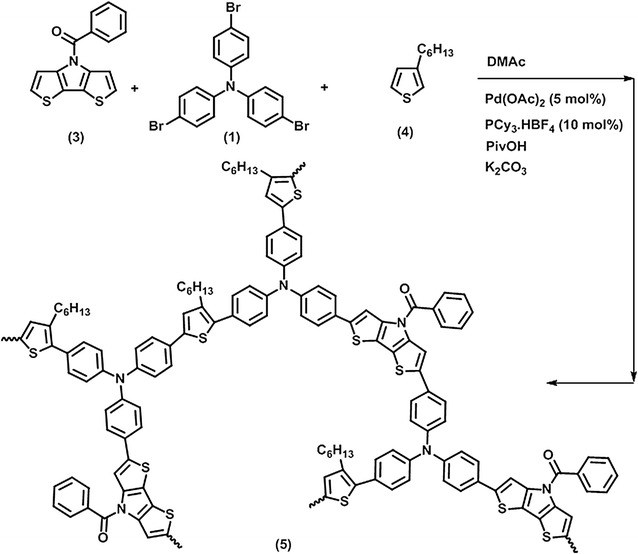
Direct arylation polycondensation of *N*-benzoyl dithieno[3,2-b:2′,3′-d]pyrrole, 3-hexylthiophene and tris(4-bromophenyl)amine monomers, resulting in PBDP3HTTPA

**Scheme 2:**
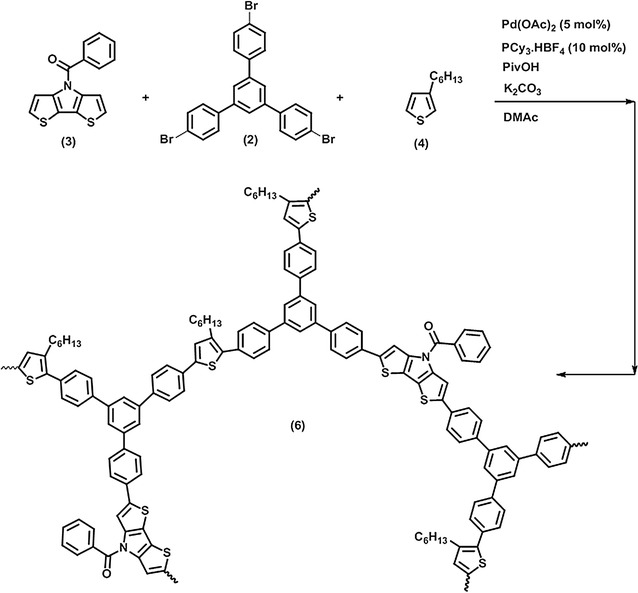
Direct arylation of polycondensation of *N*-benzoyl dithieno[3,2-b:2′,3′-d]pyrrole monomers, 3-hexylthiophene and 1,3,5-tris(4-bromophenyl)benzene monomers, resulting in PBDP3HTTPB

Two hyperbranched polymers having *N*-benzoyl dithieno[3,2-b:2′,3′-d]pyrrole and 3-hexylthiophene monomer units linked with triphenylamine or triphenylbenzene as chain extender, named as PBDP3HTTPA and PBDP3HTTPB, respectively, were aimed to be synthesized. Their synthesis pathways are illustrated in Schemes 1 and 2, respectively.

### Monomer synthesis

Tris(4-bromophenyl)amine was synthesized via bromination using *N*-bromosuccinimide, according to a procedure previously reported [38]. On the other hand, 1,3,5-tris(4-bromophenyl)benzene was synthesized from 4-bromoacetophenone using H_2_SO_4_ (conc.) and K_2_S_2_O_7_ as the catalytic system following the procedure reported by Prasad et al. [39]. *N*-benzoyl dithieno[3,2-b:2′,3′-d]pyrrole (monomer 3) was prepared via an amidation reaction by using copper(I) iodide and DMEDA as the catalytic system in the presence of K_2_CO_3_ at the reflux temperature for 24 h [15].

**Fig. 1:**
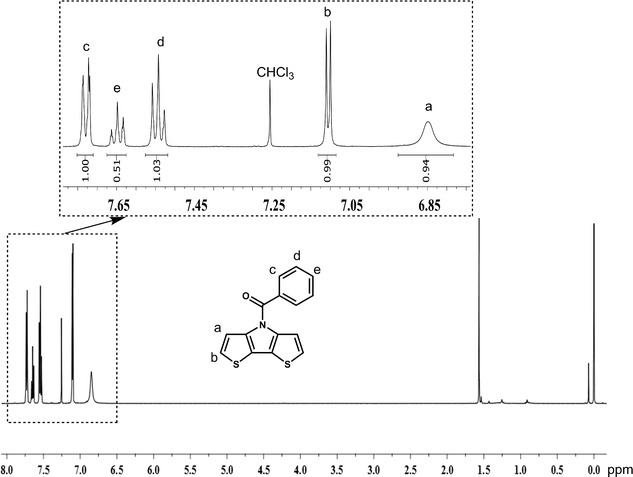
^1^H NMR spectrum of *N*-benzoyl dithieno[3,2-b:2′,3′-d]pyrrole (monomer 3)

The structure of monomer 3 was determined via ^1^H NMR. The ^1^H NMR spectrum of monomer 3 (Fig. 1) shows a doublet peak at 7.73 ppm (peak c), a triplet peak at 7.65 ppm (peak e, Fig. 1) and a triplet peak at 7.55 ppm (peak d) corresponding to the protons of the benzene ring. The doublet peak at 7.1 ppm (peak b) and the singlet peak at 6.85 ppm (peak a) are assigned to the protons of the thiophene rings. The presence of these peaks, along with their integral ratios, indicate that the amidation reaction has taken place successfully to give the *N*-benzoyl dithieno[3,2-b:2′,3′-d]pyrrole monomer.

### Direct arylation polycondensation

**Fig. 2:**
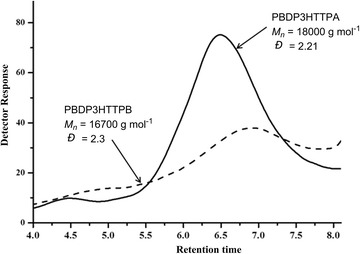
GPC traces of hyperbranched conjugated polymers PBDP3HTTPA (solid line) and PBDP3HTTPB (dash line)

The chemical structures of hyperbranched conjugated polymers PBDP3HTTPA and PBDP3HTTPB and corresponding monomers are shown in Schemes 1 and 2, respectively. The monomers *N*-benzoyl dithieno[3,2-b:2′,3′-d]pyrrole (3) and 2,5-dibromo-3-hexylthiophene (4) underwent direct arylation polycondensation with tris(4-bromophenyl)amine (1) (or 1,3,5-tris(4-bromophenyl)benzene (2)) to build hyperbranched conjugated polymer structures. The polycondensation was carried out in the DMAc solvent at 100 °C with Pd(OAc)_2_ and PCy_3_.HBF_4_ as the catalyst and ligand, respectively. The PBDP3HTTPA hyperbranched polymer was synthesized by polymerization of a mixture of monomers (1), (3) and (4), the solution of which became dark orange after 2 h, and gradually turned into black accompanying the appearance of a solvent-insoluble black solid. After 24 h, the hyperbranched polymer was obtained by purification via extraction, filtration via a Celite layer to remove the Pd catalyst, subsequent washing and precipitation in cold *n*-heptane. On the other hand, the polymerization mixture of monomers (2), (3) and (4) showed a red color in 3 h after initiation, which then gradually changed into dark red. The obtained PBDP3HTTPB was purified in a similar way to PBDP3HTTPA. The yield of both reactions were in the range of 80–90% after 24 h. It should be noted that the solvent-insoluble part (about 5%) and soluble oligomer fraction were removed via filtration through Celite layer and via washing with acetone, respectively. The number average molecular weights (M_n_s) as determined by GPC relative to polystyrene standards of PBDP3HTTPA and PBDP3HTTPB were 18,000 and 16,700 g mol^−1^, with polydispersities of 2.1 and 2.3, respectively (Fig. 2, Table 1). These hyperbranched conjugated polymers were soluble well in common organic solvents such as chloroform, THF, toluene, DMAc and insoluble in methanol, diethyl ether and *n*-heptane.

**Table 1:** Characteristics of hyperbranched conjugated polymers prepared via direct arylation polycondensation of monomers 1, 3 and 4 (PBDP3HTTPA)^a^, and of monomers 2, 3 and 4 (PBD3HTTBP)^b^

Entry	Polymer	Temp (^o^C)	Yield (%)^c^	M_n_ (g mol^−1^)^d^	M_w_/M_n_^d^	3HT: BD: TPA (TPB) molar ratio^e^ (*r*)
1	PBDP3HTTPA	100	82	18,000	2.1	1:1.18:1.45
2	PBDP3HTTPB	100	90	16,700	2.3	1:1.38:1.59

^a^Conditions: [1]_0_ = 44 mM; [3]_0_ = [4]_0_ = 33 mM; [Pd(OAc)_2_] = 1.6 mM; [PCy_3_.HBF_4_]_0_ = 3.0 mM; [PivOH]_0_ = 30 mM

^b^Conditions: [2]_0_ = 44 mM; [3]_0_ = [4]_0_ = 33 mM; [Pd(OAc)_2_] = 1.6 mM; [PCy_3_.HBF_4_]_0_ = 3.0 mM; [PivOH]_0_ = 30 mM

^c^After removal of chroloform-insoluble and acetone-soluble fractions

^d^Determined by GPC with THF as eluent and polystyrene calibration

^e^Molar ratio between 3-hexylthiophene, *N*-benzoyl dithieno[3,2-b:2′,3′-d]pyrrole and triphenylamine (or triphenylbenzene) units calculated by ^1^H NMR, based on the integration ratio between peak f at 2.6 ppm and o at 7.7 ppm (Fig. 2a) for P3HT3HTTPA, and the integration between peak f and overlapping shift range of peaks l, m and n around 7.75 ppm for P3HT3HTTBP

### Polymer structure

The polymer structures were characterized by transmission FT-IR and ^1^H NMR spectroscopies. The FT-IR spectra of PBDP3HTTPA and PBDP3HTTPB displayed several bands between 2850 and 3060 cm^−1^ asigned to CH stretching modes of *n*-hexyl groups and ring C–H stretching vibrations. The bands at 1585 and 1492 cm^−1^ are ascribed to the aromatic C=C stretching and aromatic C–H deformation vibrations, respectively, while the bands at 1323 and 1274 cm^−1^ are assigned to the C–N stretching of triphenylamine units. The appearance of a strong absorption band at 1700 cm^−1^ indicates the existence of C=O group of the *N*-benzoyl dithieno[3,2-b:2′,3′-d]pyrrole moiety in the polymer structure. The bands at 696 and 628 cm^−1^ are ascribed to the thiophene C–S–C bending and S–C stretching vibrations, respectively.

**Fig. 3:**
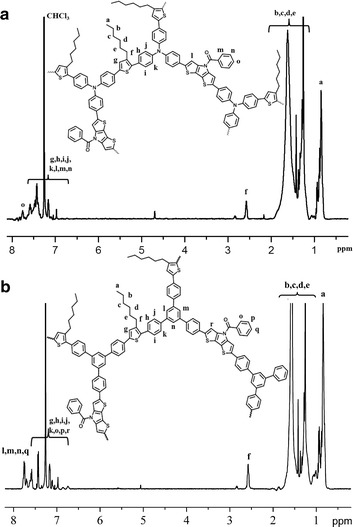
^1^H NMR spectra of PBDP3HTTPA (**a**) and PBDP3HTTPB (**b**) in CDCl_3_

In the ^1^H NMR spectrum of hyperbranched conjugated polymer PBDP3HTTPA (Fig. 3a), a signal was observed 7.65 ppm (peak o) assignable to the phenyl proton in the para position of the *N*-benzoyl dithieno[3,2-b:2′,3′-d]pyrrole moiety. The peaks from 6.85 ppm to 7.60 ppm are attributed to the aromatic protons of triphenylamine and thiophene units. Moreover, the ^1^H NMR spectrum of PBDP3HTTPA showed all characteristic peaks of the 3-hexylthiophene, triphenylamine, and *N*-benzoyl dithieno[3,2-b:2′,3′-d]pyrrole repeating units. Similarly, the ^1^H NMR spectrum of PBDP3HTTPB (Fig. 3b) also showed all characteristic peaks of the 3-hexylthiophene, triphenylbenzene and *N*-benzoyl dithieno[3,2-b:2′,3′-d]pyrrole repeating units. These results indicate that direct arylation coupling polymerization successfully took place to form the expected polymers. Additionally, we noted clearly the disappearance of the signal at 7.35 ppm in the spectrum of PBDP3HTTPA, which was originally aromatic protons closest to bromide in tris(4-bromophenyl)amine (compound 1). Similarly, the signal at 7.51 ppm disappears in the spectrum of PBDP3HTTPB, which was originally aromatic protons closest to bromide in 1,3,5-tris(4-bromophenyl)benzene (compound 2). These suggest that all three bromide groups of compound 1 and 2 were consumed, suggesting the formation of hyperbranched structures.

To reach more insights into the polymer structures, the unit ratio of 3-hexylthiophene (3HT) versus *N*-benzoyl dithieno[3,2-b:2′,3′-d]pyrrole (BD) was calculated based on the integration values of the thiophene-CH_2_ proton signal at 2.6 ppm (peak f, Fig. 2a) and the benzoyl ortho proton signal of *N*-benzoyl dithieno[3,2-b:2′,3′-d]pyrrole at 7.7 ppm (peak o, Fig. 3a). Taking into account that the molar ratio between the total number of 3HT and BD units versus the number of TPA units is 1.5, a compositional molar ratio (*r*) between BD, 3HT and TPA units of 1:1.18:1.45 was determined. In the case of PBDP3HTTPB, *r* was calculated based on the integration ratio between the thiophene-CH_2_ proton signal at 2.6 ppm (peak f, Fig. 3b) and the overlapping shift range of aromatic proton signals around 7.75 ppm of BD (peak q corresponding to 1 proton, Fig. 2b) and triphenylbenzene (peak l, m, n corresponding to 3 protons, Fig. 3b) moieties, taking into acount the molar ratio between the total number of 3HT and BD units versus the number of TPB units being 1.5. PBDP3HTTPB had a compositional molar ratio (*r*) between BD, 3HT and TPB units of 1:1.38:1.59. The characteristics of the obtained hyperbranched conjugated polymers are presented in Table 1. However, we could not determine the degree of branching by the use of ^1^H NMR integration, since the chemical shifts of branching, terminal, and linear units could not be differentiated.

In addition to the NMR results, which indirectly confirm the formation of hyperbranched structures, controlled experiments were also performed. Accordingly, one reactive site of the monomer 3-hexylthiophene (monomer 4) was blocked with a carbaldehyde (–CHO) group to give in 3-hexylthiophene-2-carbaldehyde. Direct arylation reaction between 3-hexylthiophene-2-carbaldehyde and tris(4-bromophenyl)amine (compound 1) was then conducted. Attributed to the non-participation of the carbaldehyde group in the direct arylation reaction, no hyperbranched structure was obtained, as indicated by the low molecular weight (below 1000 g mol^−1^) of the product determined by GPC and mass spectroscopic analysis. The ^1^H and ^13^C NMR results also indicated a corresponding star-structure formed from 3-hexylthiophene-2-carbaldehyde and tris(4-bromophenyl)amine. These results suggest that a hyperbranched structure could only be generated with the participation of both reactive sites of the monomer.

It should also be noted that in other controlled experiments, the direct arylation reaction between tris(4-bromophenyl)amine and N-benzoyl dithieno[3,2-b:2′,3′-d]pyrrole provided a polymer product with a poor solubility, suggesting that a hyperbranched structure was formed. On the other hand, the direct arylation reaction between tris(4-bromophenyl)amine and 3-hexylthiophene resulted in a polymer product with M_n_ of around 15,000 g mol^−1^ and Đ of 2.1.

### Thermal properties

**Fig. 4:**
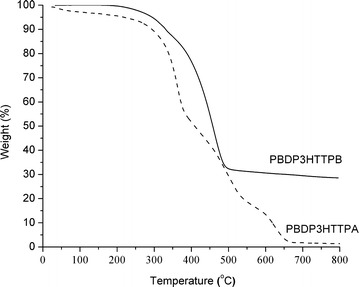
TGA curves of PBDP3HTTPA and PBDP3HTTPB

The thermal properties of hyperbranched PBDP3HTTPA and PBDP3HTTPB were investigated by thermogravimetric analysis (TGA) and differential scanning calorimetry (DSC). TGA under nitrogen flow was used to evaluate the thermal stability of the purified hyperbranched conjugated polymers in the range from room temperature to 800 °C. PBDP3HTTPA exhibited good thermal stability with decomposition temperature (5% weight loss) around 250 °C (see Fig. 4). The TGA diagram of PBDP3HTTPB showed a mass loss of 5 wt% at 300 °C as the threshold for thermal decomposition, and a loss of about 70 wt% at 500 °C.

**Fig. 5:**
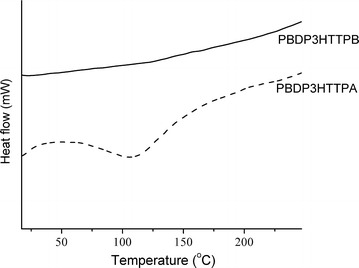
Second-heating run DSC curves (exo up) of PBDP3HTTPA and PBDP3HTTPB DSC was performed under nitrogen atmosphere at a heating rate of 10 °C min^−1^

The second heating run DSC diagram in the range from 0 to 250 °C of the conjugated hyperbranched polymers are shown in Fig. 5. No transition in this temperature range was detected for PBDP3HTTPB, while a relatively broad endotherm ascribed to a melting peak at 110 °C was observed for PBDP3HTTPA. It is well-known that linear poly(3-hexylthiophene) and poly(dithieno[3,2-b:2′,3′-d]pyrrole) chains are generally stiff with very strong intermolecular π-π stacking interactions, resulting in high melting temperatures normally above 200 °C [40–42]. Thus, the branching structure hindered the stacking of polymer chains, leading to crystalline domains with less ordered packing and so a low melting temperature range in comparison with the linear analogous polymers. On the other hand, because of the planar structure of TPB units, PBDP3HTTPB exhibits a higher oder of chain stacking than PBDP3HTTPA with propeller-like TPA moieties.

### Solid structure

**Fig. 6:**
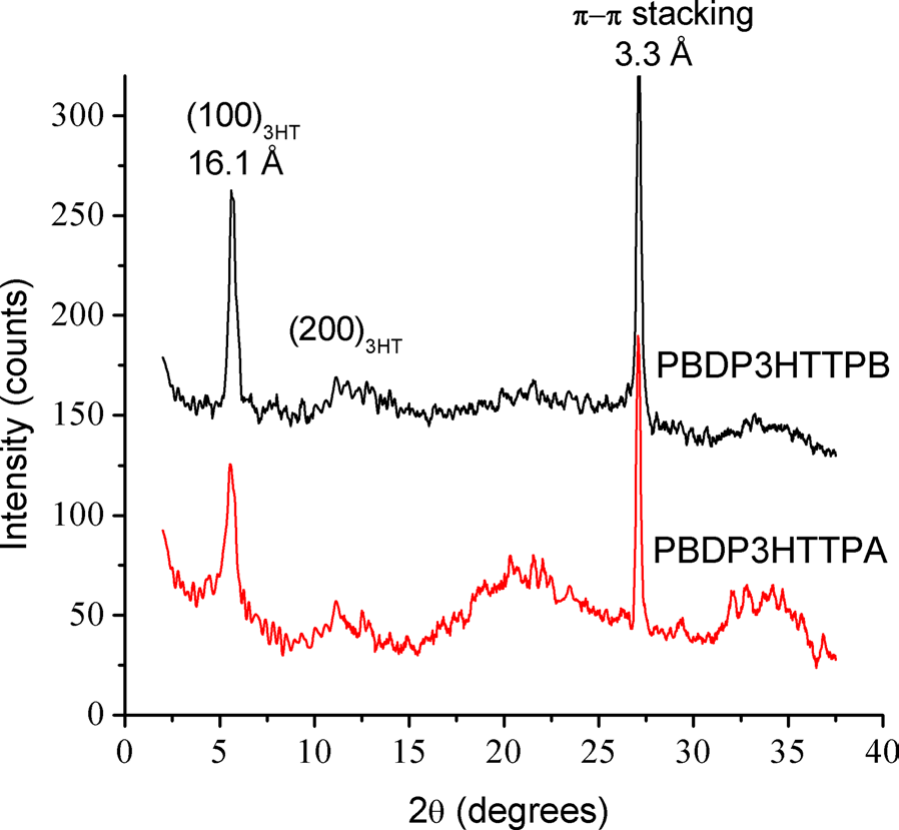
X-ray diffraction (XRD) patterns of PBDP3HTTPA and PBDP3HTTPB powders

The photophysical properties of conjugated polymers are affected by their nanostructures of solid state films. Therefore, the molecular ordering of the PBDP3HTTPA and PBDP3HTTPB hyperbranched polymers in the solid state was investigated by powder X-ray diffraction (XRD) measurements (Fig. 6). The XRD patterns of PBDP3HTTPA and PBDP3HTTPB exhibit two distinctive diffractions at 2θ = 5.5° and 27.0°, corresponding respectively to an interchain *d*-spacing of 16.1 Å between neighboring polymer chains separated by *n*-hexyl side chains [40, 43] and a π–π stacking distance of 3.3 Å. This π–π stacking distance is slightly smaller than that observed for classical poly(3-hexylthiophene) [44, 45] and is close to that observed for dithieno[3,2-b:2′,3′-d]pyrrole-based oligomers and polymers [42, 46]. Because of the difference in the planar geometry of TPB and TPA units, PBDP3HTTPB exhibits a slightly higher ordered packing, indicated by the somewhat higher intensities of diffraction peaks. In addition, the XRD pattern of PBDP3HTTPA shows a broad amorphous halo centered ca. 21° associated with scattering from a disordered packing of *n*-hexyl side chains [47, 48] whereas this amorphous diffraction is less prominent for PBDP3HTTPB.

### Optical properties

**Fig. 7:**
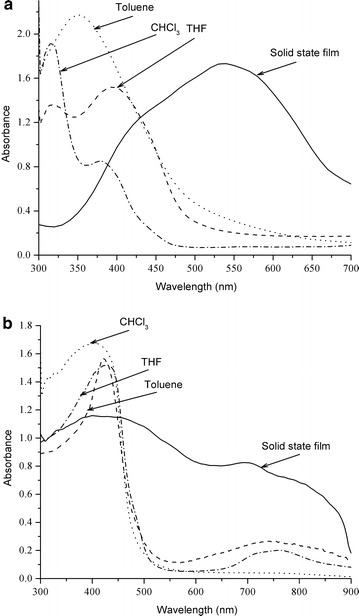
Absorption spectra of PBDP3HTTPA (**a**) and PBDP3HTTPB (**b**) in different solvents and solid state films

Figure 7a, b present the UV–vis spectra of PBDP3HTTPA and PBDP3HTTPB, respectively, measured in different solvents including CHCl_3_, THF, toluene and in solid state films. PBD3HTTPA showed an absorption maximum at 360 nm in toluene, an absorption maximum at 310 nm and a shoulder peak at 360 nm in CHCl_3_, and a maximum at 400 nm and a shoulder peak at 310 in THF, indicating slightly different conformations in different solvents. It exhibited a strong red shift in the film with absorption peak at 550 nm, reflecting a higher structural order in thin film.

In the case of PBDP3HTTPB, an absorption maximum at around 410–420 nm was observed in CHCl_3_, THF as well as toluene. However, an addition absorption peak at 750 nm was found for PBDP3HTTPB in THF and toluene, indicating the co-existence of a small fraction of polymer molecules in a more aggregated form. In solid film, besides an absorption maximum at 410 nm, PBDP3HTTPB exhibited an absorption peak at 700 nm, broadening to 850 nm. This reveals that PBDP3HTTPB has a high aggregation degree than PBDP3HTTPA in the solid state, which is in agreement with the DSC and XRD results.

**Fig. 8:**
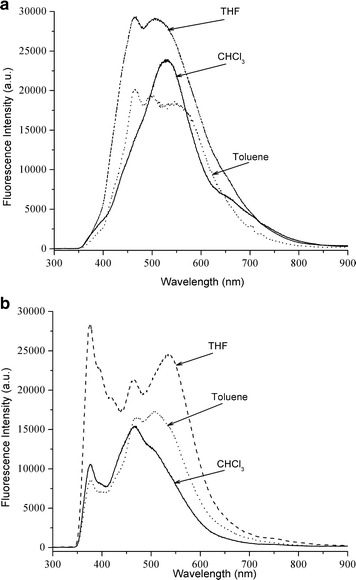
Fluorescence spectra of PBDP3HTTPA (**a**) and PBDP3HTTPB (**b**) (concentrations of 0.05 g L^−1^)

The photoluminescent (PL) spectra of the hyperbranched conjugated polymers in solutions excited at the absorption maxima are shown in Fig. 8a, b. In CHCl_3_, PBD3HTTPA displayed an emission peak at 545 nm upon excitation at 310 nm, whereas in toluene solution, PBD3HTTPA exhibited triplet peaks at 460, 500 and 560 nm upon excitation at 360 nm. In THF solution, PBD3HTTPA exhibited double peaks at 450 and 500 nm upon excitation at 400 nm. In the case of PBDP3HTTPB upon excitation at 410 nm, the polymer showed peaks at 380 nm and 475 nm in CHCl_3_, whereas it displayed triplet peaks at 380, 475 and 520 nm in both THF and toluene.

The fluorescence quantum yields (ϕ_*F*_) of the polymers in dilute CHCl_3_ were measured in comparison to 9,10-diphenylanthracence as a standard (in cyclohexane ϕ_*F*_ = 0.9), and the results are summarized in Table 2. The quantum yields increased from 0.57 for PBDP3HTTPA to 0.62 for PBDP3HTTPB. It is likely that the stronger π–π stacking effect in the structure of PBDP3HTTPB led to a higher quantum yield as a result of a lower nonradiative decay rate [49, 50].

**Table 2:** UV–vis absorption and fluorescence emission maximum wavelengths, and the fluorescence quantum yields (ϕ_*F*_) of PBDP3HTTPA and PBDP3HTTPB

Solvent	PBDP3HTTPA			PBDP3HTTPB		
	UV (nm)	PL (nm)	ϕ_*F*_	UV (nm)	PL (nm)	ϕ_*F*_
CHCl_3_	310, 360	545	0.57	410	380, 475	0.62
THF	310, 400	450, 500		420, 750	380, 475, 520	
Toluene	360	460, 500, 560		420, 750	380, 475, 520	
Film	550			410, 710		

### Solvent-induced aggregation

**Fig. 9:**
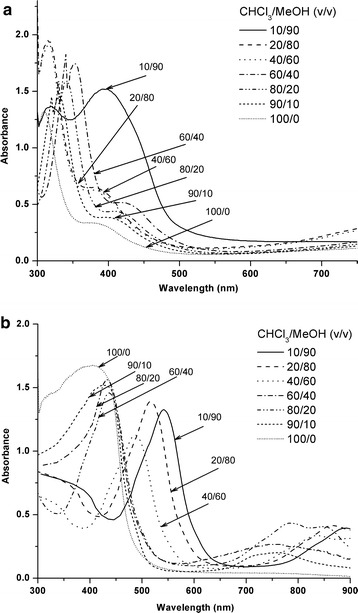
UV–vis spectra of PBDP3HTTPA (**a**) and PBDP3HTTPB (**b**) measured in CHCl_3_/MeOH mixtures

The aggregation state induced by intermolecular interactions based on π-stacking affects strongly the optical properties of conjugated polymers [51]. In solution, the H-aggregates (with parallel aligned transition dipoles) and J-aggregates (with head-to-tail aligned transition dipoles) show distinct changes in the absorption band, i.e. bathochromic (red) shifts or hypsochromic (blue) shifts, respectively, compared to the monomeric species [52, 53]. Molecular aggregation can possibly be induced by addition of a non-solvent to a polymer solution. Figure 9 shows the absorption spectra of the PBDP3HTTPA and PBDP3HTTPB hyperbranched polymers, measured in CHCl_3_/methanol mixtures. The π–π* absorption band of PBDP3HTTPA was located at 310 nm in pure CHCl_3_, indicating the coil conformation of polymer chains. The addition of methanol from 10 to 90% to polymer solution induced slight bathochromic shifts, indicating conformational changes toward the formation of aggregates. A similar effect was observed for PBDP3HTTPB when adding small amounts of methanol from 10 to 40%. Moreover, at higher methanol contents, strong red shifts were observed, indicating significant chain aggregation. Correspondingly, the absorption maximum of P3HTBTTPA shifted to 500, 520 and 550 nm at methanol contents of 60, 80 and 90%, respectively. These results also confirm that PBDP3HTTPB exhibits a higher tendency to form aggregate than PBDP3HTTPA.

## Conclusions

We have demonstrated the successful synthesis of novel hyperbranched conjugated polymers containing *N*-benzoyl dithieno[3,2-b:2′,3′-d]pyrrole and 3-hexylthiophene monomer units, linked with the triphenylamine or triphenylbenzene moiety (PBDP3HTTPA and PBDP3HTTPB, respectively), via direct arylation polycondensation in 80–90% yields. The molecular weights of the obtained polymers were 18,000 g mol^−1^ for PBDP3HTTPA and 16,700 g mol^−1^ for PBDP3HTTPB. Both polymers exhibited high structural order in thin films, which can be promising for organic solar cell applications. The UV–vis absorption of PBDP3HTTPB containing triphenylbenzene as branching unit was red-shifted as compared with PBDP3HTTPA, as a result of a higher chain packing degree. Generally, the results proved that the optical properties of these hyperbranched conjugated polymers could be controlled via alteration of the branching unit, which is useful for potential application as optoelectronic materials.

## Experimental

### Materials

3-Hexylthiophene (3HT) was purchased from TCI (Tokyo, Japan). triphenylamine, benzo [c] [1,2,5] thiadiazole, tetrahydrofuran (99.9%) and *N*-bromosuccinimide were purchased from Acros Organics. Palladium(II) acetate (Pd(OAc)_2_) (98%), tricyclohexylphosphine tetrafluoroborate (97%, PCy_3_·HBF_4_), 3,3′dibromo-2,2′bithiophene, benzamide, *N*,*N′*-dimethylethylenediamine (85%, DMEDA) and pivalic acid (PivOH) were purchased from Sigma-Aldrich. Potasium acetate (KOAc), sodium carbonate (99%), magnesium sulfate (98%), and copper iodine (CuI) were purchased from Acros and used as received. Chloroform (CHCl_3_, 99.5%), toluene (99.5%), and dimethylacetamide (DMAc, 99%) were purchased from Fisher/Acros and dried using molecular sieves under N_2_. Dichloromethane (99.8%), *n*-heptane (99%), methanol (99.8%), ethyl acetate (99%) and diethyl ether (99%) were purchased from Fisher/Acros and used as received.

### Characterization

^1^H NMR spectra were recorded in deuterated chloroform (CDCl_3_) with TMS as an internal reference, on a Bruker Avance 300 MHz. Fourier transform infrared (FT-IR) spectra, collected as the average of 64 scans with a resolution of 4 cm^−1^, were recorded from KBr disk on the FT-IR Bruker Tensor 27. Size exclusion chromatography (SEC) measurements were performed on a Polymer PL-GPC 50 gel permeation chromatograph system equipped with an RI detector, with tetrahydrofuran as the eluent at a flow rate of 1.0 mL min^−1^. Molecular weight and molecular weight distribution were calculated with reference to polystyrene standards. UV–vis absorption spectra of polymers in solution and polymer thin films were recorded on a Shimadzu UV-2450 spectrometer over a wavelength range of 300–700 nm. Fluorescence spectra were measured on a HORIBA IHR 325 spectrometer. Differential scanning calorimetry (DSC) measurements were carried out with a DSC 204 F1—NETZSCH instruments under nitrogen flow (heating rate 10 °C min^−1^). Thermogravimetric analysis (TGA) measurements were performed under nitrogen flow using a STA 409 PC Instruments with a heating rate of 10 °C min^−1^ from ambient temperature to 800 °C. Wide-angle powder X-ray diffraction (XRD) patterns were recorded at room temperature on a Bruker AXS D8 Advance diffractometer using Cu-Kα radiation (k = 0.15406 nm), at a scanning rate of 0.05 degrees per second. The data were analyzed using DIFRAC plus Evaluation Package (EVA) software. The *d*-spacing was calculated from peak positions using Cu-Kα radiation and Bragg’s law.

### Synthesis of tris(4-bromophenyl)amine (1)

*N*-bromosuccinimide (2.17 g, 12.2 mmol) and triphenylamine (1 g, 4.08 mmol) were added to anhydrous THF (10 mL) at 0 °C under nitrogen. The mixture was stirred at 50 °C for 1.5 h. After completion of the reaction, 10 mL of distilled water was added to the reaction mixture, which was extracted with dichloromethane. The organic layer was washed with 10% solution of Na_2_S_2_O_3_ and 10% solution of KOH, dried over anhydrous MgSO_4_ and concentrated. The product was precipitated in cold *n*-heptane and dried under vacuum to give a white powder (R_f_ = 0.6; yield: 67%). ^1^H NMR (300 MHz, CDCl_3_), δ (ppm): 7.35 (d, 6H), 6.95 (d, 6H). ^13^C NMR (125 MHz, CDCl_3_): (ppm): 146.10, 132.42, 125.68, 116.17. MS *m/z* (M^+^) 478. Analysis calculated for C_18_H_12_Br_3_N: C, 45.1; H, 2.51; Br, 49.49; N, 2.92. Found: C, 45.35; H, 2.41; Br, 49.35; N, 2.89.

### Synthesis of 1,3,5-tris(4-bromophenyl)benzene (2)

4-Bromoacetophenone (5 g, 25.13 mmol), 0.25 mL of H_2_SO_4_ (conc.) and K_2_S_2_O_7_ (6.6 g, 26.14 mmol) were heated at 180 °C for 16 h under a nitrogen atmosphere. The resulting crude solid was cooled to room temperature and refluxed in 25 mL of dry ethanol (EtOH) for 1 h and then cooled to room temperature. The solution was filtered and the resulting solid was refluxed in 25 mL of H_2_O to give a pale yellow solid that was then filtered. The crude product was dried under vacuum giving 7.5 g of dried product, which was recrystallized from CHCl_3_ (yield 55%). ^1^H NMR (300 MHz, CDCl_3_), (ppm): 7.51 (d, 6H), 7.60 (d, 6H), 7.68 (s, 3H). ^13^C NMR (125 MHz, CDCl_3_): (ppm): 139.82, 137.60, 130.23, 122.72, 121.43. MS *m/z* (M^+^) 539. Analysis calculated for C_24_H_15_Br_3_: C, 53.34; H, 2.77; Br, 43.89. Found: C, 53.25; H, 2.69; Br, 44.06.

### Synthesis of *N*-benzoyl dithieno[3,2-b:2′,3′-d]pyrrole monomer (BD) (3)

To a 50 mL rounded-bottomed flask equipped with a magnetic stirrer was added copper iodide (0.19 g, 1 mmol), DMEDA (1.728 mL, 8 mmol), potassium carbonate (4.15 g, 30 mmol) in the nitrogen atmosphere. Then, toluene and a small amount of distilled water (1 equiv.) were added to the reaction mixture and the solution was stirred for 30 min. Benzamide (12 mmol) was added, followed by 3,3′-dibromo-2,2′-bithiophene (3.24 g, 10 mmol). The reaction mixture was stirred for 24 h at 110 °C. The reaction was cooled to the room temperature, then washed with distilled water (3 × 20 mL) and extracted with chloroform (3 × 20 mL). The organic phase was dried by anhydrous K_2_CO_3_. The solvent was removed by rotary evaporation. The crude product was purified by silica column chromatography (eluent: heptane/ethyl acetate: 4/1) to give the isolated product as a white crystalline solid (3.82 g, R_f_ = 0.75, yield: 45.3%). ^1^H NMR (500 MHz, CDCl_3_), δ (ppm) 7.73 (d, 2H), 7.65 (t, 1H), 7.55 (t, 2H), 7.1 (d, 2H), 6.85 (s, 2H). ^13^C NMR (125 MHz, CDCl_3_): (ppm): 167.0, 143.1, 134.5, 132.4, 128.7, 124.4, 121.8, 116.4; MS *m/z* [MNa]^+^: 306.04.

### Synthesis of hyperbranched polymer based on *N*-benzoyl dithieno[3,2-b:2′,3′-d]pyrrole, 3-hexylthiophene and tris(4-bromophenyl)amine monomer moieties (PBDP3HTTPA) (5)

In a glove box, 28.34 mg (0.1 mmol) of *N*-benzoyl dithieno[3,2-b:2′,3′-d]pyrrole, 64.27 mg (0.133 mmol) of tris(4-bromophenyl)amine and 16.83 mg (0.1 mmol) of 3-hexylthiophene were dissolved in 3 mL of DMAc. To the solution, 1.03 mg (0.0048 mmol) of Pd(OAc)_2_, 3.46 mg (0.009 mmol) of PCy_3_.HBF_4_, 9.43 mg (0.09 mmol) of PivOH and 38.3 mg of K_2_-CO_3_ were added to the monomer solution. The vial was sealed with a rubber cap and then removed from the glove box. The vial was heated in a 100 °C oil bath for 24 h. After being cooled to room temperature, the reaction mixture was diluted with 30 mL of chloroform. The obtained organic layer was passed through Celite to remove the Pd catalyst and the insoluble polymer fraction, subsequently washed with 10% solution of Na_2_S_2_O_3_ and distilled water, dried over Na_2_CO_3_, concentrated and finally poured into a large amount of cold *n*-heptane to precipitate the polymer. The resulting polymer was isolated by filtration, washed with acetone to remove oligomers, and finally dried under reduced pressure at 50 °C for 24 h. A yield of 82% was obtained. FT-IR (cm^−1^): 3057, 2925, 2852, 1700, 1585, 1492, 1436, 1323, 1273, 1182, 1116, 1026, 825, 750, 721, 696, 606, 628, 542. ^1^H NMR (500 MHz, CDCl_3_), δ (ppm) 7.73 (d, 12H), 2.65 (s, 2H), 0.8–1.95 (m, 11H). ^13^C NMR (125 MHz, CDCl_3_): 167.0; 143.3, 141.0, 135.8, 132.7, 129.6, 128.7, 127.0, 126.2, 124.4, 122.1, 116.4, 32.1, 30.7, 29.0, 22.5, 14.0. GPC: *M*_*n*_ = 18,000 g mol^−1^. Đ = *M*_*w*_ */M*_*n*_ = 2.1

### Synthesis of hyperbranched polymer based on N-benzoyl dithieno[3,2-b:2′,3′-d]pyrrole, 3-hexylthiophene and 1,3,5-tris(4-bromophenyl)benzene monomer moieties (PBDP3HTTPB) (6)

In a glove box, 28.34 mg (0.1 mmol) of *N*-benzoyl dithieno[3,2-b:2′,3′-d]pyrrole, 72.45 mg (0.133 mmol) of 1,3,5-tris(4-bromophenyl)benzene and 16.83 mg (0.1 mmol) of 3-hexylthiophene were dissolved in 3 mL of DMAc. To the solution, 1.03 mg (0.0048 mmol) of Pd(OAc)_2_, 3.46 mg (0.009 mmol) of PCy_3_.HBF_4_, 9.43 mg (0.09 mmol) of PivOH and 38.3 mg of K_2_-CO_3_ were added to the monomer solution. The vial was sealed with a rubber cap and then removed from the glove box. The vial was heated in a 100 °C oil bath for 24 h. After being cooled to room temperature, the reaction mixture was diluted with 30 mL of chloroform. The obtained organic layer was passed through Celite to remove the Pd catalyst and the insoluble polymer fraction, subsequently washed with 10% solution of Na_2_S_2_O_3_ and distilled water, dried over Na_2_CO_3_, concentrated and finally poured into a large amount of cold *n*-heptane to precipitate the polymer. The resulting polymer was isolated by filtration, washed with acetone to remove oligomers, and finally dried under reduced pressure at 50 °C for 24 h. A yield of 90% was obtained. FT-IR (cm^−1^): 3059, 2917, 2851, 1700, 1584, 1560, 1490, 1436, 1319, 1274, 1183, 1117, 1011, 825, 753, 721, 696, 628, 542. ^1^H NMR (500 MHz, CDCl_3_), δ (ppm) 7.85–6.9 (d, 13H), 2.65 (s, 2H), 0.8–1.95 (m, 11H). ^13^C NMR (125 MHz, CDCl_3_): 167.0; 143.3, 141.0, 135.8, 131.5, 129.0, 127.3, 120.2, 124.4, 122.1, 116.4, 32.1, 30.7, 29.0, 22.5, 14.0. GPC: *M*_*n*_ = 16,700 g mol^−1^. Đ = *M*_*w*_ */M*_*n*_ = 2.3.

### Synthesis of 3-hexylthiophene-2-carbaldehyde (for controlled experiment)

3-Hexylthiophene-2-carbaldehyde was synthesized according to the procedures reported in the literature [54, 55] with some modification. 3-Hexylthiophene (1 g) was dissolved in 100 mL of anhydrous toluene under nitrogen. DMF (4.6 mL, 59.2 mmol) and phosphorus(V)oxychloride (POCl_3_) (4.91 mL, 58 mmol) were then added to the solution. The reaction was performed at 75 °C for 24 h. The solution was cooled down to room temperature, followed by the addition of a saturated aqueous solution of sodium acetate. The solution was stirred for 4 h. Then, the compound was extracted with CHCl_3_ and dried over MgSO_4_. Then the solution was filtered and evaporated to obtain a crude compound. Finally, the crude compound was purified over silica column with hexane/ethyl acetate (v/v: 5/95) as eluent (R_f_ = 0.8, 0.9 g). The yield was 77.6%. ^1^H NMR (500 MHz, CDCl_3_), δ (ppm): 9.01 (s, 1H), 7.55 (d, 1H), 6.92 (d, 1H), 2.85 (t, 2H), 1.59 (m, 2H), 1.23 (m, 6H), 0.81 (t, 3H). ^13^C NMR (125 MHz, CDCl_3_), δ (ppm): 182.1, 152.8, 138.0, 134.6, 130.5, 31.6, 31.2, 29.0, 28.6, 22.6, 14.0. MS *m/z* (M^+^) 196, Analysis calculated for C_11_H_16_OS: C, 67.30; H, 8.22; O, 8.15; S, 16.33. Found: C, 66.73; H, 9.05; O, 7.85; S, 16.37.

### Direct arylation reaction between 3-hexylthiophene-2-carbaldehyde and tris(4-bromophenyl)amine (controlled experiment)

Direct arylation reaction between 3-hexylthiophene-2-carbaldehyde and tris(4-bromophenyl)amine was performed, resulting in star-shaped 5,5′,5″-(nitrilotris(benzene-4,1-diyl))tris(3-hexylthiophene-2-carbaldehyde). Procedure: 0.1 g (0.51 mmol) of 3-hexylthiophene-2-carbaldehyde and 82.15 mg (0.17 mmol) of tris(4-bromophenyl)amine were dissolved in 20 mL DMAc. To the solution, 5.5 mg (0.025 mmol) of Pd(OAc)_2_, 19.22 mg (0.05 mmol) of PCy_3_.HBF_4_, 52.4 mg (0.5 mmol) of PiOH and 212 mg of K_2_CO_3_ were added to the monomer solution. The vial was sealed with a rubber cap and was freeze–pump–thaw degassed for several times. Then the reaction was heated in a 100 °C oil bath for 24 h. After being cooled to room temperature, the reaction mixture was diluted with 100 mL of chloroform, washed with brine three times and dried over MgSO_4_. The obtained organic layer was passed through Celite to remove the Pd catalyst, concentrated and finally purified over silica column with hexane/ethyl acetate eluent (v/v: 20/80) (R_f_ = 0.7, 113 mg) to give the isolated product as a dark yellow solid. The yield was 80.1%. ^1^H NMR (500 MHz, CDCl_3_), δ (ppm): 10.1 (s, 1H), 7.60 (d, 6H), 7.13 (s, 3H), 6.9 (d, 6H), 2.6 (t, 6H), 1.59 (m, 6H), 1.33 (m, 18H), 0.91 (t, 9H). ^13^C NMR (125 MHz, CDCl_3_), δ (ppm): 181.7, 152.4, 147.2, 141.0, 127.2, 125.3, 31.6, 29.7, 29.4, 28.0, 22.6, 14.1. MS m/z (M^+^) 828.4, Analysis calculated for C_51_H_57_NO_3_S_3_: C, 73.96; H, 6.94; N, 1.69; O, 5.80; S, 11.61. Found: C, 73.46; H, 6.81; N, 1.70; O, 6.60; S, 11.43.
